# Japan's regulations on the experimental use of wild-caught Japanese macaques and its historical context

**DOI:** 10.1016/j.crneur.2026.100171

**Published:** 2026-10-25

**Authors:** Gyo Nakao, Shu Ishida, Masanori Kataoka, Chie Kobayashi, Tamami Fukushi, Tomonori Nakamura, Tsutomu Sawai

**Affiliations:** a Uehiro Division for Applied Ethics, Graduate School of Humanities and Social Sciences, Hiroshima University, Higashi-Hiroshima, Japan; b Faculty of Human Welfare, Department of Human Welfare, Tokyo Online University, Japan; c Research Centre on Emerging Technology and Governance, Niigata University, Japan; d Institute for the Advanced Study of Human Biology (ASHBi), Kyoto University, Kyoto, Japan; e Department of Anatomy and Cell Biology, Graduate School of Medicine, Kyoto University, Kyoto, Japan; f Hakubi Center for Advanced Research, Kyoto University, Kyoto, Japan; g Graduate School of Humanities and Social Sciences, Hiroshima University, Higashi-Hiroshima, Japan; h Centre for Biomedical Ethics, Yong Loo Lin School of Medicine, National University of Singapore, Singapore

**Keywords:** Animal experiment, Wildlife, Primate, Macaque, Regulation, Ethics, History

## Abstract

Non-human primates (NHPs) have long been indispensable to drug discovery and biomedical research, particularly in fields such as neuroscience and immunology. At the same time, because many NHP species are threatened with extinction, the use of wild-caught NHPs in research has become increasingly restricted internationally, and laboratory NHPs are supplied primarily through captive breeding. Against this global backdrop, Japan occupies a distinctive position. While it maintains a breeding-based supply system for Japanese macaques (*Macaca fuscata*) used in research, much larger numbers of wild Japanese macaques are captured and euthanized each year under legally authorized wildlife management programs. From the perspective of cumulative animal suffering, the parallel operation of these two systems appears paradoxical. This article reviews Japan's current regulations on the experimental use of wild-caught macaques, explains historically how the present situation emerged, and considers the ethical implications for internationally harmonized regulations.

## Highlights

• Each year Japan captures over 20,000 wild macaques, most of which are killed.
• Experimental use of wildlife-management macaques is tightly restricted in Japan.
• This policy reflects a conservation priority on local populations, not species.
• Positions on this issue diverge sharply depending on the ethical values prioritized.
• Global NHP rules should be context-sensitive, not one-size-fits-all.

## Graphical abstract

**Figure:**
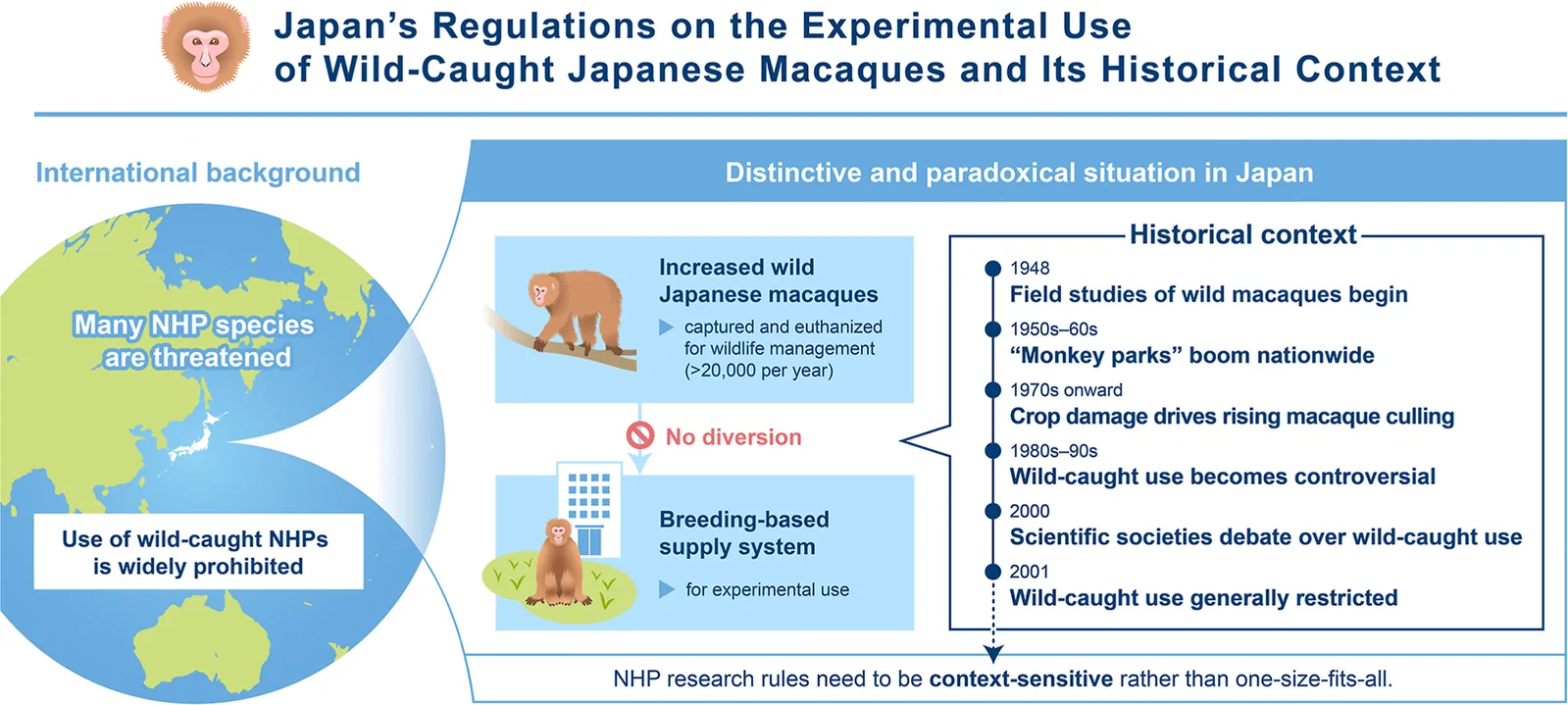

## 1 Introduction

Non-human primates (NHPs) are among the most important laboratory animals in basic biological research—including neurobiology—as well as in medical research and the evaluation of pharmaceutical efficacy and safety. Their importance derives from their close genetic, anatomical, physiological, and behavioral similarities to humans (National Academies of Sciences, Engineering, and Medicine, 2023).

In recent years, however, maintaining a stable supply of NHPs for research has become increasingly difficult as demand has grown, particularly for infectious disease studies. Macaques (genus *Macaca*), the most commonly used NHPs, are distributed across much of East, Southeast, and South Asia, as well as parts of Africa. Many countries, including the United States and members of the European Union, depend heavily on imports. China, formerly the largest exporter, halted macaque exports during the COVID-19 pandemic, and this halt remains in place. As a result, prices have risen and supply has become increasingly unstable worldwide (Grimm, 2023). Under these conditions, there is a growing need for an internationally coordinated regulatory framework that can secure access to NHPs for research while addressing animal welfare and related ethical concerns.

One notable contribution to this effort is the article by Hartig et al. (2023), which systematically compares regulations and procedures for neuroscience research involving NHPs across 13 countries, using 27 items focused primarily on animal ethics and welfare.

One of the items Hartig and colleagues use for comparison is whether the research use of wild-caught NHPs is permitted. Permitting such use might increase the number of animals available for research, although it would leave significant technical challenges unresolved (notably the risk of zoonotic infection). However, the research use of wild-caught NHPs raises not only ethical concerns related to animal welfare but also challenges related to biodiversity. According to the IUCN Red List, roughly 60% of primate species are currently classified as Threatened, and demand for biomedical research has been recognized as one factor contributing to biodiversity loss (Estrada et al., 2017; Fernández et al., 2022). In addition, recent supply pressures have reportedly been linked to illegal capture and trafficking of threatened wild primates, posing serious conservation risks (Gamalo et al., 2024). From these perspectives, restrictions on the research use of wild-caught NHPs can be considered rational. Hartig and colleagues report that in 11 of the 13 countries they examined (i.e., all except India and the United States), the use of wild-caught NHPs for research is banned. For example, the EU prohibits the use of wild-caught animals in scientific procedures in principle and requires NHPs used in research to be captive-bred or sourced from self-sustaining colonies, subject to scientifically justified exceptions (European Parliament and Council of the European Union, 2010, Arts. 9–10). What is of interest to us here is that Japan is included amongst these 11 countries.

This description of Japan is technically correct but warrants additional explanation because the situation regarding local NHPs in Japan is distinctive in terms of biodiversity. Japan's only native NHP species, the Japanese macaque (*Macaca fuscata*), is classified as Least Concern on the Red List (Watanabe and Tokita, 2020), and population growth and crop damage are widely discussed problems. As part of administrative wildlife management, more than 20,000 wild Japanese macaques are captured each year, and presumably killed. Notably, until the 1990s, a portion of these captured macaques was transferred to research institutions and used in scientific and medical experiments. Subsequent regulatory changes, however, ended this practice: wild macaques captured for culling are no longer used for research in contemporary Japan.

Japan therefore presents an unusual—and seemingly paradoxical—regulatory situation. Large numbers of wild Japanese macaques are captured through wildlife-management programs and subsequently euthanized, while Japanese macaques used in biological or medical research are supplied through a separate captive-breeding system. How did these two systems come to coexist? Why did Japan come to exclude macaques captured for wildlife management from experimental use while simultaneously establishing a separate breeding-based supply system for research?

This article addresses this question by first examining the current regulatory framework governing Japanese macaques captured for wildlife management and then reconstructing the historical process through which that framework emerged, with particular attention to the policy controversy around 2000.

## 2 Methods

This study combines a review of currently operative regulations with a documentary reconstruction of their historical formation. It is not a systematic review, and no single bibliographic database defines its corpus. For the current regulatory framework (Section 3), we examined the relevant Japanese statutes, materials published by the Ministry of the Environment (MOE), the prefectural Category II Specified Wildlife Management Plans for Japanese macaques (obtained from the official websites of the respective prefectural governments), guidelines issued by the relevant academic societies, and the regulations and guidelines adopted by major primate-research institutions.

For the historical reconstruction (Section 4), we drew primarily on contemporaneous sources, including statements and reports issued by academic societies and articles published in journals such as *Primate Research* and *Experimental Animals*. These materials were supplemented by later retrospective accounts and memoirs. Retrospective sources were read critically and, where accounts differed, greater evidentiary weight was given to contemporaneous documentation. We also examined newspaper coverage, particularly articles published in the *Asahi Shimbun*, which reported extensively on issues concerning Japanese macaques during the period examined. Articles were identified through the Asahi Shimbun Cross-Search database using combinations of Japanese-language search terms such as “Japanese macaque,” “experiment,” and “Wildlife Protection Act.” All translations from Japanese are our own.

## 3 Current regulations

In contemporary Japan, a wild Japanese macaque may be captured under three principal pathways, each legally distinct, provided by the Wildlife Protection and Management Act, the country's principal wildlife statute: capture for damage prevention (to residential environments, agriculture, forestry, fisheries, and ecosystems), capture for population adjustment, and capture under an academic-research permit. The first two together constitute “wildlife management” and are carried out primarily by municipalities and prefectural governments through two prefecture-level instruments: each prefecture's Wildlife Protection and Management Plan and its Category II Specified Wildlife Management Plan (Ministry of the Environment, Japan, 2017).

The third pathway, academic-research capture, is legally separate and requires case-by-case authorization. In FY2020, 166 Japanese macaques were captured for academic research purposes under prefecture-issued permits and 4 under MOE-issued permits, for a total of 170 (Ministry of the Environment, Japan, n.d.). Available public statistics do not indicate whether these animals were used in invasive laboratory experiments, in observational or ecological studies, in veterinary research, or in other forms of academic research, nor do they report their subsequent disposition. These figures should therefore not be read as evidence that all the 170 wild macaques entered biological or medical experimentation.

The present article focuses primarily on macaques captured for wildlife management, which are far more numerous than those captured under academic-research permits. In FY2020, 21,985 Japanese macaques were captured for damage prevention and 6062 were captured for population adjustment (Ministry of the Environment, Japan, n.d.).

Nevertheless, MOE, which administers the Wildlife Protection and Management Act, has indicated that macaques captured for wildlife management should not be used for biomedical research. Its *Guidelines for Preparing Specified Wildlife Protection and Management Plans (Japanese Macaque Edition)* instruct that captured individuals “should be euthanized using methods that minimize suffering (e.g., administration of anesthetics or gunshot) and should not be used as experimental animals” (Ministry of the Environment, Japan, 2024).

Following these Guidelines, prefectures formulate Category II Specified Wildlife Management Plans for Japanese macaques. As of August 2026, 29 prefectures had established such plans. Of these, 15 explicitly state that captured macaques should or must not be used for experiments, require that they be killed, in most cases specifying euthanasia (i.e., methods that minimize suffering), or contain both types of provision (Supplementary Table 1). Where capture is conducted pursuant to these plans, diversion to research is effectively precluded.

The great majority of Japanese macaques captured for wildlife management are, we infer, killed in accordance with these Guidelines. A 2004 MOE report based on a survey of the relevant prefectures stated that captured macaques were “mostly” disposed of by killing, with only limited cases of captive holding (Ministry of the Environment, Japan, 2004). More recent prefectural data suggest that this pattern has continued: in Kanagawa Prefecture in FY2025, all 42 macaques recorded as captured for wildlife management were killed (Kanagawa Prefecture, 2026). Comprehensive nationwide data on the post-capture disposition of macaques are not currently available, but the sources that do exist strongly indicate that killing remains the predominant outcome.

In addition to governmental instruments, academic societies have issued guidance that, while not legally binding, functions as influential “soft law” in practice. In its *Guidelines for the Care and Use of Laboratory Nonhuman Primates (2013)*, the Primate Society of Japan restricts the experimental use of wild Japanese macaques as follows:

From the standpoint of conserving wild primate populations in Japan, in principle, Japanese macaques of wild origin should not be used in animal experiments. Japanese macaques must not be captured for the purpose of experimental use. Instead, it is desirable to use offspring obtained from breeding stock maintained in appropriate facilities to supply macaques for research purposes. (Primate Society of Japan, 2013)

Similarly, the Japan Neuroscience Society states in the *Guidelines for the Care and Use of Nonhuman Primates in Neuroscience Research* that “[e]ach institution must legally introduce laboratory macaques with known birth and management status that have been bred for use in experiments and research” (Japan Neuroscience Society, 2021).

Major domestic primate research institutions have adopted comparable principles. For example, the National Institutes of Biomedical Innovation, Health and Nutrition (which houses the Tsukuba Primate Research Center) states in its *Basic Principles for Experiments Using Monkeys* that “in principle, the monkeys used in experiments must be limited to individuals bred and reared specifically for experimental use” (National Institutes of Biomedical Innovation, Health and Nutrition, 2023). Kyoto University's Center for the Evolutionary Origins of Human Behavior likewise states in its *Guidelines for the Care and Use of Primates* that “wild monkeys, even when obtained through harmful wildlife control, are in principle not accepted, so as not to encourage easy expansion of culling” (Center for the Evolutionary Origins of Human Behavior, Kyoto University, 2024).

Taken together, these measures mean that, although not explicitly prohibited by statute, the experimental use of wild Japanese macaques in Japan is precluded in practice.

## 4 Historical context

The policy direction described above solidified only after the year 2000. Until then, a portion of wild macaques captured under nuisance control were transferred to universities and other research institutions and used in experiments. This section outlines the historical process that led to the current situation. Supplementary Table 2 summarizes the major milestones.

After World War II, two research communities in Japan turned their attention to wild Japanese macaques. One was the Experimental Animal Research Association, organized mainly by medical researchers in Tokyo, which explored the feasibility of using Japanese macaques at scale as laboratory animals (Tajima, 1984; Nomura and Iinuma, 1991). The other was the Primate Research Group, led by Kinji Imanishi at Kyoto University, which sought to study macaque society as a window onto human evolution.

In 1952, the Primate Research Group achieved what is often described as the world's first case of sustained provisioning (artificial feeding) of wild macaques, on Kōjima Island, Miyazaki Prefecture. Provisioning enabled close-range observation of otherwise wary animals, made individual identification feasible, and opened the way to systematic study of social structure—developments that became foundational for Japanese primatology (Sugiyama, 2020).

These provisioning practices also contributed to the emergence of “monkey parks*,*” a characteristically Japanese hybrid of research and tourism. Monkey parks used provisioning to attract macaques to specific sites so that humans could observe them at close range. They were expected to serve three functions: (1) entertainment for visitors, (2) field sites for primatologists, and (3) supply points for experimental animals (Setoguchi, 2013). Following the opening of Takasakiyama Monkey Park in 1953 in Oita, monkey parks expanded rapidly; by the 1960s, more than 30 existed domestically (Mito, 1995). Over time, many regions came to host semi-free-ranging, provisioned macaque groups that moved between park areas and surrounding mountains under partial human management.

Initially, the Experimental Animal Research Association and the Primate Research Group collaborated to promote park creation, establish the Japan Monkey Centre as a national hub for macaque research and supply, and form a federation of monkey parks. They envisioned a nationwide network that would connect parks and channel “surplus” animals into experimental use. In practice, however, the system proved difficult to implement, and purposeful removals to supply experimental animals remained limited (Wada, 2008).

Over time, many parks experienced overpopulation driven by continued provisioning. Habituation associated with provisioning reduced macaques' wariness toward humans. Combined with rural depopulation in Japan's hilly and mountainous areas, expanding macaque populations caused severe agricultural damage across multiple localities. Furthermore, as visitor numbers declined from around 1965 onward, parks also lost the financial capacity to manage these problems (Mito, 1995). This was followed by a gradual increase in macaque captures conducted as “harmful wildlife control” (nuisance control) under the Wildlife Protection Act, the predecessor of the Wildlife Protection and Management Act (Fig. 1). In parallel, younger generations of field primatologists increasingly criticized provisioning as a form of ecological disturbance (Wada, 1989).

**Fig. 1:**
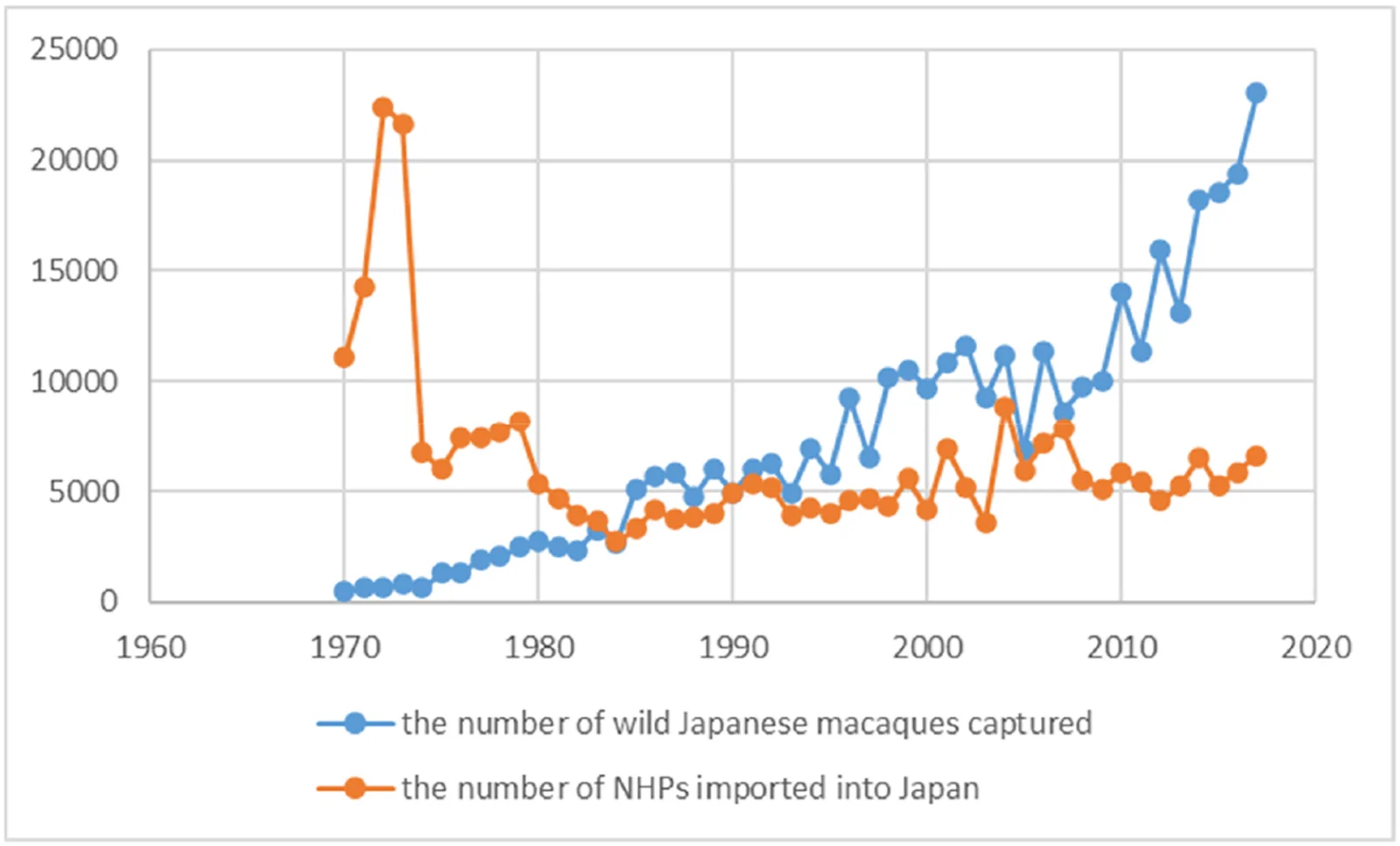
**Historical statistics on reported wild Japanese macaque captures and NHP imports into Japan.** Capture figures are reported by fiscal year and import figures by calendar year. The two series are not directly comparable and the figure is not intended to demonstrate a causal relationship. Import figures cover all NHPs and are not limited to macaques or animals imported for experimental purposes. Capture data were obtained from the Japan Hunters Association (n.d.). Import data were taken from the Committee for the Study of Experimental Primate Resources and Development (1987) for years up to 1986; from the December issues of *Nihon Bōeki Geppyō* [*Monthly Return of the Trade of Japan*] for 1987–2001; and from the Ministry of Finance, Japan (n.d.) for 2002 onward (searched using HS code 0106.11). Differences among these sources may affect the continuity of the series.

Meanwhile, laboratory-based researchers faced tightening access to experimental NHPs. In Japan, primate use expanded after the development and introduction of a domestically produced live polio vaccine in the early 1960s, and large numbers of cynomolgus and rhesus macaques were imported. However, the adoption of the Convention on International Trade in Endangered Species of Wild Fauna and Flora in 1973, together with India's restrictions on—and eventual prohibition of—rhesus macaque exports in the 1970s, made overseas procurement increasingly difficult and led to a reduction in NHP imports (Science Council of Japan, 1978). Available Japanese statistics on NHP imports and wild Japanese macaque captures are shown in Fig. 1 as contextual information. Because the two datasets differ in temporal units, sources, coverage, and classification, no direct quantitative comparison between them is attempted here.

Against this backdrop, demand rose for Japanese macaques as a substitute for imported animals. Some Japanese macaques captured through nuisance control were transferred to universities and other research institutions, and their use in experiments increased over time (Chiba, 1980; Wada, 2008). Minezawa et al. (1986) reported the results of a questionnaire survey of Japanese research institutions, including the mortality data reproduced in Table 1, and concluded from the survey as a whole that the use of Japanese macaques had increased as access to rhesus macaques became more restricted. The survey also showed that Japanese macaques were used across a wide range of research areas, particularly in neuroscience and physiology.

**Table 1:** **Reported deaths of selected NHP species at Japanese research institutions, based on a 1985 questionnaire survey.** Adapted from table 11 of Minezawa et al. (1986). The survey received responses from 160 of 252 institutions selected as likely users of primates. Respondents reported deaths among primates used in their laboratories, including deaths from disease and accidents. These figures therefore represent responding institutions only and are neither national totals nor direct measures of experimental use. “1977 and earlier” is an aggregate category and is not directly comparable with the annual counts for 1978–1984.

	1977 and earlier	1978	1979	1980	1981	1982	1983	1984
Cynomolgus macaques	830	342	474	387	379	289	298	390
Rhesus macaques	791	45	51	73	49	93	56	32
Japanese macaques	157	69	68	115	109	84	135	160
NHPs total	1851	481	627	632	590	557	690	687

Younger field-based primatologists and conservation NGOs, however, regarded this trend as risky. They argued that, in the absence of adequate population surveys, unnecessary culling could be justified under the banner of securing experimental animals. Moreover, they emphasized that conservation should be pursued not only at the species level but also at the level of local populations and their genetic composition. For field researchers in particular, the loss of local populations would also mean the loss of study populations (Iwano, 1978; Committee for the Study of Experimental Primate Resources and Development, 1992).

In the 1990s, agricultural damage caused by wildlife—including macaques, bears, boars, and deer—worsened. In response to demands from agricultural stakeholders nationwide, agriculture-oriented members of the National Diet (Japan's parliament) intensified calls to loosen restrictions on culling through revision of the Wildlife Protection Act (Sugimoto, 1998). To reconcile deregulation pressures with conservation demands, the Environment Agency (later reorganized as MOE) introduced the concept of *wildlife management*—placing wildlife populations under human control—and submitted a revision bill to the Diet in February 1999, which passed in June. The revision enabled each prefecture to prepare Specified Wildlife Protection and Management Plans for populations that had markedly increased or decreased, and to adjust population sizes accordingly (Hayama, 2019).

In July 1999, following the revision of the Wildlife Protection Act, the Environment Agency established the Committee on Wildlife Protection and Management to deliberate related guidelines and standards. Its 14 members consisted primarily of specialists in fields such as wildlife management, ecology, and nature conservation. They included a primatologist specializing in the conservation ecology of Japanese macaques who also served on the board of the Primate Society of Japan. By contrast, the committee included no physiologists, neuroscientists, or other members who could represent the interests of researchers using Japanese macaques as experimental animals (Environment Agency, Japan, 1999a). When the Environment Agency solicited public comments in September 1999 on the Standards for the Eighth Wildlife Protection Plan, three board members of the Primate Society of Japan, having obtained information from the aforementioned primatologist about the committee's internal deliberations, submitted an opinion statement warning against overly easy adoption of nuisance control and requested language such as: “As a general rule, live-captured individuals should not be used as experimental animals” (Nigi et al., 2000).

Reflecting these views, the committee discussed limiting the forms of scientific research recommended as beneficial uses of captured animals to research directly related to wildlife protection and management, thereby excluding experimental research in fields such as medicine. The standards were subsequently revised along these lines (Environment Agency, Japan, 1999b; Yoshida, 2001). The draft version of the *Technical Manual for Specified Wildlife Protection and Management Plans (Japanese Macaque Edition)* subsequently released by the Environment Agency stated that “captured individuals should, in principle, be euthanized” and that “capture for the purpose of experimental use is not permitted.”

Laboratory-based researchers who relied on NHPs—particularly in physiology, neuroscience, and medicine—strongly objected (Asahi Shimbun, 2000). According to members of the Physiological Society of Japan, many such researchers were not directly involved in policy discussions, which largely took place within the Environment Agency's sphere (Ezure and Kaneko, 2002). From October to November 2000, the Physiological Society of Japan submitted three statements arguing that banning the experimental use of captured wild Japanese macaques would increase procurement costs and ultimately weaken research capacity in Japan. The Society also argued that limited biological resources should be used efficiently, and contended that euthanizing captured macaques while sourcing different macaques for experiments would increase overall animal deaths and conflict with the principles of animal welfare (Committee on Animal Experiments, The Physiological Society of Japan, 2001).

In November 2000, the Primate Society of Japan issued a statement in the name of its Board of Directors adopting the position that “the conservation of wild Japanese macaques should be the first priority,” and signaling a critical stance toward experimental use. It argued that nuisance control was being implemented too readily and suggested that the prospect of experimental use could function merely as a pretext. The Society further expressed concern that “if efforts are made to meet the growing demand for experimental monkeys, many local populations of wild Japanese macaques will be subjected to even greater disturbance.” Emphasizing “international trends,” it also warned that refraining from experiments using wild-caught monkeys was becoming an international norm and that research using wild individuals would face increasing difficulty in gaining international acceptance (Primate Society of Japan, 2000). Throughout, the Society consistently prioritized ecosystem conservation over the protection of individual animals.

The Primate Society of Japan's conservation-first stance was also evident in its response to an emerging issue in Wakayama Prefecture, where non-native populations of Taiwanese macaques had formed and ecologists warned of genetic disturbance through hybridization with Japanese macaques. In 2001, the Society submitted a petition to the Governor of Wakayama requesting that the prefecture capture and euthanize all Taiwanese macaques and their hybrids, and offered its cooperation. Although some local residents opposed euthanasia and supported alternatives such as sterilization and captive holding, the Society argued that such measures would delay action and urged prompt capture and euthanasia (Primate Society of Japan, 2001).

The position of the Primate Society of Japan on the experimental use of wild macaques was also shaped by the activities of a “third” group of actors. Even among laboratory-based researchers, some had long argued that Japan should discontinue reliance on wild-caught animals and instead establish a breeding center for Japanese macaques intended for research. Wild-caught macaques posed multiple challenges as laboratory animals: they could carry zoonotic pathogens such as B virus, their ages were often uncertain, individual variation was substantial, and acclimation to laboratory conditions was difficult. Particularly in biomedical fields, many researchers therefore preferred purpose-bred animals. At the same time, as the establishment of a breeding center required substantial resources, proponents repeatedly encountered the counterargument that wild-caught macaques were more economical (Honjō, 2013). This created a convergence of interests between these proponents and field-based primatologists (Noguchi, 1987). A biomedical researcher, after the founding of the Primate Society of Japan in 1985, sought cooperation with field-based researchers and advocated that the Society adopt an ethical code, drafting an initial proposal (Honjō, 1986; Sugiyama et al., 2020). As a result, the Society's 1986 *Guiding Principles for Animal Experiments Using Nonhuman Primates* included the statement that experimental monkeys should be animals bred and reared under human control (Primate Society of Japan, 1986).

Media coverage around 2000 sharpened the conflict, reporting that captured wild macaques ostensibly released to Takasakiyama had in fact been transferred to a medical university, and that others were sold through unlicensed dealers. Laboratory-based scientists using monkeys came to be viewed as lacking due attention to conservation and as failing to manage animals appropriately, and they faced intense criticism and accusations from animal welfare organizations (Asahi Shimbun, 1999; Asahi Shimbun, 2000; Shimizu, 2000).

Ultimately, at the end of 2000, the Environment Agency published the final version of the Technical Manual, which contained provisions calling for the euthanasia of captured wild Japanese macaques and prohibiting their experimental use. In response, the Physiological Society of Japan and the Japan Neuroscience Society shifted toward a more conciliatory approach. Coordinating with the Primate Society of Japan, they submitted a petition to the Ministry of Education, Culture, Sports, Science and Technology (MEXT) and other authorities, calling for the establishment of a hub to breed and supply Japanese macaques for research (Isa, 2004). The outcome was a policy trade-off: prohibition of experimental use of captured wild macaques was paired with the establishment of a breeding-based supply system. The initiative was implemented under MEXT's National BioResource Project (NBRP), launched in FY2002, and subsequent efforts have focused on building a breeding and distribution system to provide a stable supply of high-quality, habituated Japanese macaques that are safer with respect to infectious diseases (Higashino et al., 2016).

Even this project, however, faced criticism from animal welfare organizations. Because a breeding colony first had to be established, the project initially sought to accept Japanese macaques from zoos and similar facilities facing overbreeding. These plans met strong opposition from anti–animal experimentation organizations: one facility withdrew from a planned transfer, and one local government that had approved transfers faced lawsuits by residents seeking to halt them (Asahi Shimbun, 2003; Asahi Shimbun, 2004). In response, the NBRP sought to address criticism by promoting appropriate animal experimentation through measures such as formulating guidelines, establishing review systems, conducting training courses, and issuing licenses. A report by key project members also identified the near disappearance of experiments using wild-caught Japanese macaques as one of the project's achievements (Nakamura et al., 2017).

Since the pilot phase began in FY2006, the NBRP has distributed a total of 1248 individual Japanese macaques to research institutions through FY2025, corresponding to an annual mean of about 62 over 20 years (Kyoto University NBRP “Japanese macaques,” 2026). This level of supply is relatively modest when compared with the situation in the early 2000s—when Japanese neuroscientists reportedly used approximately 270 Japanese macaques per year (2001–2003)—and to the annual supply target of 200 at the launch of the NBRP (Isa et al., 2009). This contrast is even more pronounced when viewed against the scale of wildlife management captures, which now exceed 20,000 wild Japanese macaques per year. While the research use of Japanese macaques is currently not necessarily the “mainstream” of animal experimentation in Japan, the primates most commonly used are cynomolgus macaques, heavily utilized by contract research organizations and pharmaceutical manufacturers, with annual use estimated at no fewer than 4000 individuals. Within academia, marmosets and cynomolgus macaques are widely used (Miyoshi et al., 2024).

## 5 Discussion: ethical and regulatory implications

This article has outlined the current regulatory landscape and its historical context regarding the experimental use of wild-caught Japanese macaques. Importantly, the process of establishing the policy precluding the experimental use of wildlife-management macaques does not appear to have been accompanied by sufficiently exhaustive deliberation among core stakeholders. The review committee convened by the Environment Agency included a board member of the Primate Society of Japan, but it included no one positioned to represent the interests of researchers who used captured macaques in experiments. Those who opposed the emerging regulation voiced their objections through written statements, yet there is no evidence that they were able to attend the Environment Agency's meetings on the matter. Moreover, a close reading of the debate indicates that the opinion statements submitted by the Physiological Society of Japan raised ethically significant issues—such as the possibility that the prohibition could result in the killing of a greater number of macaques—yet the Environment Agency reached its decision before those issues were examined in depth.

Does the maintenance of this restriction, which continues to limit the experimental use of wild macaques today, in fact lead to more animals being killed overall? If appropriately screened wildlife-management animals could substitute for some purpose-bred research animals without increasing capture incentives, the current system—which authorizes the euthanasia of culled wild macaques while simultaneously breeding new ones for research—might paradoxically increase the total number of macaques killed by humans, thereby conflicting with the ideal of animal welfare.

Addressing this question requires examining two largely separate issues. The first is whether captured wild macaques could substitute for purpose-bred research animals. As some medical researchers have repeatedly noted, wild macaques present several difficulties as laboratory animals: their age and health history are typically unknown, they are unaccustomed to artificial environments, and they frequently carry pathogens transmissible to humans. By contrast, Japanese macaques supplied by the NBRP—with well-documented genetic backgrounds, acclimation to laboratory conditions, and reduced biosecurity risks—are in high demand for research in fields such as neuroscience and immunology. The benefit and necessity of highly controlled breeding of NHPs in these scientific fields do not hinge on whether the experimental use of wild-caught Japanese macaques is permitted. Nevertheless, in other areas such as molecular and cellular biology, basic physiology, and certain forms of preclinical research, strict acclimation or genetic uniformity is not always the primary concern. In some lines of research, genetic diversity is considered advantageous, or greater weight may be placed on securing a sufficiently large number of analyzable individuals. If safety concerns—especially zoonotic risks—could be adequately managed, wild-caught Japanese macaques could be considered as candidates for research use in such domains.

The second issue is whether relaxing the restriction on the experimental use of captured macaques would increase the number of animals captured. As some primatologists argued, integrating culled animals into research risks creating a problematic incentive structure where scientific demand could inadvertently encourage additional captures under the guise of damage control. In recent years, however, the annual number of wild Japanese macaques captured has more than doubled relative to around 2000, reaching over 20,000, whereas the NBRP supplies only a few dozen per year for research. So long as demand for Japanese macaques as laboratory animals does not greatly exceed this scale, relaxing the restriction seems unlikely to affect the number captured. That situation could change, however, if research demand were to grow well beyond the current scale, or if a substantial decline in wild populations were to sharply reduce the number of animals captured for wildlife management.

In addition, a defensible ethical evaluation of the paradox cannot rely solely on a utilitarian calculus of numerical reduction in animal deaths; it must carefully weigh the qualitative severity of welfare burdens alongside other complex scientific and social dimensions. Utilizing a wild-caught macaque already scheduled for euthanasia under wildlife management programs might seemingly reduce breeding demand. Also, it is not always clear whether the “euthanasia” of wild-caught macaques sufficiently considers their welfare, and in this regard, well-equipped research institutions may be better placed to ensure the ultimate euthanasia is carried out appropriately. However, keeping wild-caught macaques alive for research could simultaneously prolong or intensify that individual animal's suffering. Specifically, wild macaques subjected to capture, transport, quarantine, and laboratory acclimation may experience intense, prolonged psychological and physiological distress compared to purpose-bred animals who are habituated to controlled laboratory environments from birth. Furthermore, wild-caught primates carry substantial biosecurity and zoonotic risks (such as B virus), which can compromise scientific validity and data quality. This, in turn, risks violating the 3Rs principles—particularly reduction and refinement—by requiring larger sample sizes to overcome uncontrolled genetic and environmental variables. A robust and defensible governance framework must therefore address all of these dimensions: balancing the overall reduction in animal deaths against the heightened severity of individual suffering, public legitimacy, and the strict requirements of biosecurity. A detailed specification of such safeguards, however, lies beyond the scope of this article.

What this article has shown is that Japan's regulations took shape within a historical context unique to Japan. Hartig et al. (2023) highlighted broad similarities across many countries' regulations for research involving NHPs, yet they also pointed out that different, country-specific viewpoints may underlie this convergence. The Japanese case supports this insight. Japan's current rules may resemble the prevailing Western approach in their practical effect, yet they rest on a somewhat different normative foundation. They strongly prioritize the conservation of macaques at the level of local populations and genetic composition, rather than the species-level conservation that is usually the focal point of environmentally motivated considerations. At the same time, the ideal of reducing the number of animals killed, or minimizing cumulative animal suffering, has not necessarily received due attention. Then, to respect the welfare of NHPs more comprehensively, it might be preferable for Japan to adopt regulations that differ from the current ones, which are, on the surface, similar to international standards. This suggestion by no means negates the importance of the strict regulations governing the use of wild macaques for research in other regions, such as the EU. On the contrary, it intends to apply their underlying principle of respect for animal welfare within a different natural and historical context.

Similarly, in countries other than Japan, respect for animal welfare might call for regulatory approaches alternative to the existing ones. For example, on St. Kitts, African green monkeys (*Chlorocebus sabaeus*)—transported from West Africa during the colonial slave trade, free of natural predators, and now numbering on the order of the island's human population—are widely regarded as an invasive agricultural pest, yet are simultaneously valued as a tourist attraction. And, in sharp contrast to Japan, they are captured in the wild and used in biomedical experiments—both on the island and, through export, in laboratories abroad (Gallagher et al., 2022). Given this highly specific and complex situation, it would be an urgent issue to consider what can and should be done to ensure the welfare of NHPs.

As we have demonstrated, positions on the research use of wild-caught NHPs can diverge sharply depending on which values are prioritized: the lives and suffering of individual animals, ecosystem and population-level conservation, the number of experimental animals, the quality of experimental animals, and the protection of agriculture. Even a blanket prohibition—often assumed to support conservation goals—may under certain conditions increase the number of macaques killed by humans and thus conflict with the ideal of animal welfare, another important ethical value that merits serious consideration. Whether this is the case depends, among other things, on the substitutability of wildlife-management animals for purpose-bred animals and the risk that research demand might create incentives for additional capture. For future international frameworks to secure NHPs for research in a more ethically desirable manner, what is required is not a one-size-fits-all approach that would unconditionally seek convergence in formal regulation, but context-sensitive approaches that respond to the conditions under which wild primates exist in each country.

## CRediT authorship contribution statement

TS: conceptualization, funding acquisition, investigation, methodology, supervision, writing—review and editing. GN: conceptualization, funding acquisition, investigation, methodology, writing—original draft, writing—review and editing. SI: conceptualization, funding acquisition, investigation, writing—review and editing. MK: conceptualization, funding acquisition, investigation, writing—review and editing. CK: conceptualization, funding acquisition, investigation, writing—review and editing. TF: conceptualization, investigation, writing—review and editing. TN: conceptualization, funding acquisition, investigation, writing—review and editing.

## Statement of AI use

LLMs (ChatGPT-5.2) were used for translation and proofreading. This use conforms to the guidelines in Porsdam Mann, S., Vazirani, A.A., Aboy, M. et al. Guidelines for ethical use and acknowledgement of large language models in academic writing. *Nat Mach Intell* 6, 1272–1274 (2024). https://doi.org/10.1038/s42256-024-00922-7.

## Funding

This study was supported by the 10.13039/501100001691Japan Society for the Promotion of Science (JSPS) [10.13039/501100001691KAKENHI Grant Numbers 24K00039 and 25K24754 to TS; JP24K22446 and JP26K15899 to GN; JP25K16058 to MK], the Center for Collaborative Sciences, Headquarters for Co-creative Future Sciences, 10.13039/501100003790Hiroshima University [to 10.13039/100015876GN, SI, MK, 10.13039/501100001515CK, and 10.13039/100016851TS], the 10.13039/100016355Uehiro Foundation on Ethics and Education [UEHIRO2023-0141 to 10.13039/100016851TS], and Hakubi Project, 10.13039/501100005683Kyoto University [to TN].

## Declaration of competing interest

No potential conflict of interest was reported by the author(s).

## Data Availability

No data was used for the research described in the article.
