# Supplementary material for: Japan's regulations on the experimental use of wild-caught Japanese macaques and its historical context
Source: Curr Res Neurobiol. 2026 Oct 25;11:100171. doi: 10.1016/j.crneur.2026.100171 (PMC13742342; doi:10.1016/j.crneur.2026.100171)
Supplement: Supplementary file 1 [file mmc1.pdf]

LEARN MORE
Current Research in Neurobiology
Part of the CO+RE suite of journals
OPEN ACCESS

CRNEUR Peer Review Guidance

This document highlights important aspects of the review process, which may differ from other journals. We aim for our review process to be as fair and balanced as possible, as highlighted in the *editorial introduction* to *CRNEUR*.

1. Our review process is transparent. Alongside accepted papers, we publish editor, reviewer and author interactions during the peer-review process. The authors choose to submit their manuscript for a single- or double-anonymized review, either being identified (single-anonymized) or remaining anonymous (double-anonymized) during the review process. Reviewers have the option to reveal their identities during the review process or after its completion. Editors are known throughout.

2. Constructive and efficient feedback. We aim for a fair, balanced, efficient and constructive peer-review process by everyone involved, regardless of manuscript outcome. Editors follow journal guidance and will make decisions based on input from reviewers and authors (see Figure).

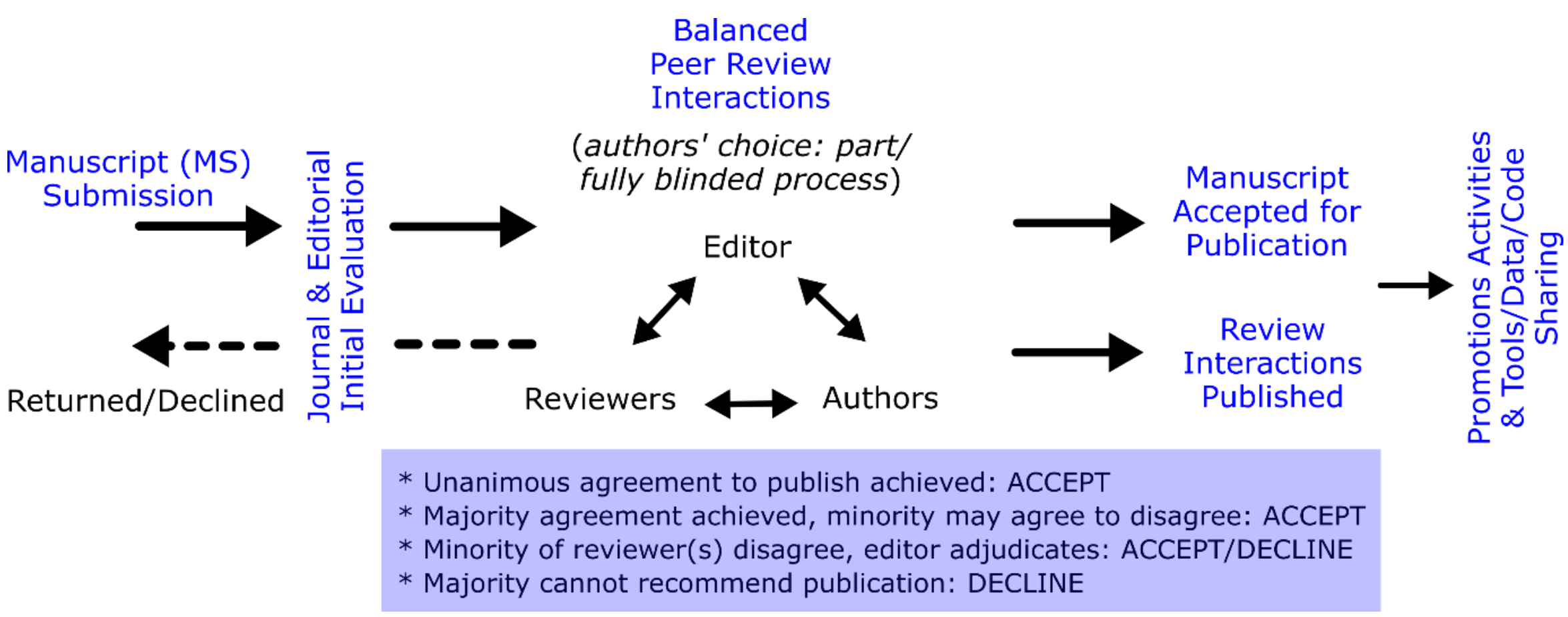

3. Author, reviewer and editor interactions during the review process. We welcome cordial and well-substantiated arguments by everyone involved in the process about whether and when the manuscript is clear and strong enough to be published. Requests for additional data and analysis need to be well justified. There may be good reasons to “agree to disagree” with an author or reviewer on what makes for the clearest and most compelling paper. With the transparent review process, it is possible for authors and reviewers to discuss these points, guided by the editor. This interaction will be published alongside an accepted manuscript.

4. Review process reports are only published for accepted papers. If your manuscript is not published in *CRNEUR*, the review process reports are available to the authors. Reports for manuscripts not accepted by *CRNEUR* are not released without the authors’ permission.

5. Rewarding reviewers. We hope that taking part in this peer-review process will be rewarding. We are exploring ways that reviewers can be rewarded for their time and effort. We encourage you to help to guide journal innovations by sharing your views as part of the *CRNEUR* survey.

Author Checklist

Please complete this Author Checklist and upload the entire document, including the preceding one page Peer Review Guidance, together with your manuscript submission. If you select 'No' to a question, please give a reason.

1. *CRNEUR author submission guidance and tips*

Please confirm that you have read the CRNEUR author guidance, in particular the Author's Guide for CRNEUR Submissions: Y/N YES

2. *Experimental design and execution*

Are the hypotheses being tested clearly stated? Y/N No
Reason: This is a non-experimental historical, regulatory, and ethical analysis rather than a hypothesis-testing study.

Are the primary outcome measures clearly stated and recorded together with their precision as defined by standard error or confidence limits/intervals? Y/N No
Reason: No primary outcome measures are applicable.

Was a power analysis conducted to determine group sample size and balance, or what was the sample size decision based on? Y/N No
Reason: No experimental group design or sample size determination was performed.

Were experiments blinded and randomized to avoid investigator bias? Y/N No
Reason: No experiments were conducted.

Do the data support the assumptions of the statistical tests (e.g., parametric tests) and were significance thresholds predetermined and corrected for multiple comparisons? Y/N No
Reason: No inferential statistical testing or multiple-comparison procedure was used.

Were the experiments independently repeated to establish within-study replicability? Y/N No
Reason: No experiments were conducted.

Were appropriate study controls in place (as examples, control conditions, vehicle controls, positive and negative compound/treatment controls) to establish study validity and effect sizes? Y/N No
Reason: No experimental controls were applicable to this study design.

3. *Research ethics statement*

Is there a clear ethics statement for human and/or animal research including information on the approval and regulatory body? Y/N No
Reason: This study did not involve any original research with human participants or animal subjects.

4. *Reagent/animal authentication/validation*

If the study involves cells, are the animal details provided complete – source, species, strain, sex, age/weight, genetic modification status? Y/N No
Reason: This study did not involve cells or experimental animals.

Were all experimental materials (reagents, compounds, antibodies, cell line/animal genotype/phenotype, analysis tools etc.) fully validated/authenticated? Y/N
No
Reason: No reagents, compounds, antibodies, cell lines, or experimental animals were used.

5. Data and Code availability

Were all data that were generated reported and discussed in the manuscript, including whether data were excluded and the reasons stated clearly? Y/N No
Reason: This is not a primary experimental data study.

Is the data and code to replicate the results shared in a community accepted repository (e.g., Open Science Framework)? Y/N No
Reason: This is not a primary experimental data study.

6. Author information

Is a conflict of interest statement for all authors included with the manuscript? Y/N YES

Have all organizations providing funding for the submitted work been listed in the Acknowledgements section? Y/N YES

Are the Author Contribution of all authors of the manuscript involved in the work clearly stated at the end of the manuscript per ICMJE guidelines (http://www.icmje.org/recommendations/browse/roles-and-responsibilities/defining-the-role-of-authors-and-contributors.html)? Y/N YES

Please contact the journal team if you have any questions: crneur@elsevier.com
