# Supplementary material for: Japan's regulations on the experimental use of wild-caught Japanese macaques and its historical context
Source: Curr Res Neurobiol. 2026 Oct 25;11:100171. doi: 10.1016/j.crneur.2026.100171 (PMC13742342; doi:10.1016/j.crneur.2026.100171)
Supplement: Supplementary file 2 [file mmc2.docx]

Peer Review Overview

**Manuscript Title:** Japan’s Regulations on the Experimental Use of Wild-Caught

Japanese Macaques and Its Historical Context

| Received |  | Jul 10, 2026 |
| --- | --- | --- |
| 1st Decision |  | Aug 22, 2026 |
| 1st Revision Submitted |  | Sep 21, 2026 |
| Accepted |  | Oct 16, 2026 |

1st Decision letter

### **Reference:** CRNEUR-D-26-00023R1

### **Title:** Japan’s Regulations on the Experimental Use of Wild-Caught Japanese Macaques and Its Historical Context

### **Journal:** Current Research in Neurobiology

Dear Prof Sawai,

Thank you for submitting your manuscript to Current Research in Neurobiology. 

I have completed my evaluation of your manuscript. The reviewers recommend reconsideration of your manuscript following major revision. I invite you to resubmit your manuscript after addressing the comments below. Please resubmit your revised manuscript by **Sep 21, 2026**. 

When revising your manuscript, please consider all issues mentioned in the reviewers' comments carefully: please outline every change made in response to their comments and provide suitable rebuttals for any comments not addressed. Please note that your revised submission may need to be re-reviewed.  

To submit a revision, go to https://www.editorialmanager.com/crneur/ and log in as an Author. You will see a menu item call Submission Needing Revision. You will find your submission record there.

When you submit the revised manuscript, please provide a separate document, uploaded as 'Detailed Response to Reviewers,' that carefully details, point-by-point, the list of changes made in response to the reviewers' comments. You may also include a suitable rebuttal to any specific request for change that you have not made. The journal strongly encourages you to submit two versions of your revised manuscript in order to facilitate the evaluation process: one with changes in the manuscript marked (upload as file type: Manuscript (REVISED, text with changes Marked)), in addition to an unmarked, production-ready version (required) (upload as file type: Manuscript).

Research Elements (optional)
This journal encourages you to share research objects - including your raw data, methods, protocols, software, hardware and more – which support your original research article in a Research Elements journal. Research Elements are open access, multidisciplinary, peer-reviewed journals which make the objects associated with your research more discoverable, trustworthy and promote replicability and reproducibility. As open access journals, there may be an Article Publishing Charge if your paper is accepted for publication. Find out more about the Research Elements journals at https://www.elsevier.com/authors/tools-and-resources/research-elements-journals?dgcid=ec_em_research_elements_email.

Current Research in Neurobiology values your contribution and I look forward to receiving your revised manuscript.

Kind regards,  

Anna S Mitchell, Ph.D. 
Editor in Chief 
Current Research in Neurobiology

**Comments from Editors and Reviewers:**

**Reviewer #1:**

Overview

This is short communication that examines why Japan prohibits the experimental use of wild-caught Japanese macaques even though more than 20,000 wild macaques are reportedly captured and euthanized annually through wildlife-management programs. The authors argue that Japan's policy developed through a distinctive historical conflict among field primatologists focused on conservation and genetic diversity, laboratory researchers concerned about primate supply and the inefficient killing of a potential resource, biomedical researchers favoring purpose-bred macaques because of biosecurity, standardization, and acclimation advantages and government agencies responding to agricultural damage, wildlife management, and public controversy. The authors conclude that Japan's present arrangement may be ethically paradoxical: purpose-bred macaques are raised and killed for research while much larger numbers of wild macaques are killed and discarded. They argue that international primate-research regulation should be sensitive to national ecological and historical circumstances rather than uniformly prohibiting wild-caught animals.

The manuscript presents an interesting, underexamined regulatory history and would be valuable to readers interested in primate research ethics, animal welfare, and science policy. The historical reconstruction is its strongest contribution. However, the principal ethical claim—that the prohibition results in more macaques being killed overall—is not established by the evidence presented. The article presently moves too quickly from a plausible ethical question to a counterfactual conclusion. I would recommend major revision.

Major concerns

1. The central premise "more animals are killed" claim is not demonstrated
The manuscript and especially the media statement imply that Japan kills more macaques because wildlife-management animals cannot be used in research. That conclusion requires evidence that wild-caught macaques would substitute for purpose-bred animals and reduce breeding or research sacrifice approximately one-for-one. The available numbers do not establish that counterfactual. Wildlife-management captures may occur regardless of research demand, and many captured animals may be unsuitable for research for a variety of reasons including unknown age and health history, infectious-disease risks, behavioral stress and difficulty acclimating, uncertain genetic or experimental characteristics, scientific-validity requirements, logistical and regulatory costs.

I suggest that the authors recast this as a question or conditional argument. For example, if appropriately screened wildlife-management animals could substitute for some purpose-bred research animals without increasing capture incentives, the current system might increase the total number of macaques killed.

2. "Ban" and "wild-caught" require more precise definitions
The paper acknowledges that Japanese law does not uniformly prohibit capturing macaques for academic research and reports 170 research-permitted captures in FY2020. The operative restriction instead appears to concern diverting macaques captured under wildlife-management authority into experimental use. The manuscript should consistently distinguish:
1. Macaques captured specifically under academic-research permits.
2. Macaques captured for damage prevention.
3. Macaques captured for population adjustment.
4. Purpose-bred research macaques.
5. Animals used in invasive experiments versus observational, ecological, veterinary, or other research.

Calling the current policy a "de facto ban on wild-caught macaques" is potentially misleading when academic-research capture remains legally possible.

3. The historical-review methodology is insufficiently described
The manuscript does not explain systematically how documents and events were identified and selected. It should include a short methods section stating; databases, archives, government websites, and date ranges searched; search terms and inclusion criteria; how the 29 prefectural plans were identified; which 15 plans expressly prohibited experimentation or required euthanasia; whether Japanese passages were translated by the authors; how potentially conflicting historical accounts were adjudicated; whether newspaper reports were used as primary evidence or contextual sources.

A supplementary table listing all 29 prefectures, plan dates, relevant provisions, and source links would materially strengthen the paper.

4. The ethical analysis needs a more balanced framework
The discussion emphasizes numerical reduction in deaths but does not adequately compare all relevant dimensions, including; total number of animals killed, duration and intensity of suffering, stress associated with capture, transport, quarantine, and laboratory acclimation, biosecurity and zoonotic risk, scientific validity and data quality, conservation of local populations and genetic structure, the possibility that research demand could create incentives for additional capture, the 3Rs, especially replacement and reduction, public legitimacy and governance safeguards.

Using a macaque already scheduled for euthanasia might reduce breeding demand, but it could also prolong or intensify that individual animal's suffering. A defensible conclusion requires considering both numbers and welfare severity.

5. The quantitative displays cannot support strong trend or causal conclusions
Figure 1 combines:
* Japanese-macaque captures reported by fiscal year.
* Imports of all nonhuman primates reported by calendar year.
* Data assembled from different sources and classification systems.

These are not directly comparable. The caption and text should emphasize their descriptive nature and explain discontinuities, missing values, category changes, and whether imported animals include species or purposes unrelated to the argument.

Table 2 also needs qualification. It reports deaths among survey respondents, not national use; "cumulative through 1977" is presented alongside annual counts, and deaths do not necessarily equal animals entering experiments. It should not be used to infer a national trend without denominators and survey-coverage information.

6. The policy recommendation needs concrete safeguards
If the authors wish to argue that limited research use of wildlife-management animals should be considered, they should specify the conditions necessary to prevent harmful incentives. Possible safeguards include:
* Capture decisions made independently of research demand.
* No payment structure that rewards additional capture.
* Prospective veterinary and infectious-disease screening.
* Independent ethical review.
* Scientific justification that purpose-bred animals are unnecessary.
* Transparent reporting of capture reason, disposition, and research use.
* Monitoring of whether use actually reduces purpose-bred demand.
* Limits by research type and welfare burden.

Without such conditions, "context-sensitive regulation" remains too general.

Minor comments
* Replace "overabundance" with more neutral wording unless a population-management assessment supports it.
* "Legally mandated wildlife-management programs" may overstate the legal position; the programs appear legally authorized and administratively implemented, not necessarily mandated in every case.
* Correct "annal supply target" to "annual supply target."
* Correct "an one-size-fits-all approach" to "a one-size-fits-all approach."
* Use consistent capitalization for "Japanese macaques."
* Clarify whether the 1,190 NBRP figure represents animals distributed, experiments, or transfers.
* The manuscript is long and detailed for a short communication. Table 1 could be moved to supplementary material and the historical narrative shortened.
* The graphical abstract and highlights reproduce the causal claim too strongly and should be revised with the manuscript.
* The phrase "ecologists' priority" risks portraying one heterogeneous community as having a single position. More qualified language would be preferable.
* The discussion of inadequate stakeholder participation should be supported with evidence about the actual consultation process, not inferred only from later objections.

**Reviewer #2:**

Overview

Short communication about paradoxical of use Japanese monkeys involved in research are taken from breeding programmes when more than 20,000 are culled each year to curb overpopulation and stop crops being decimated.
In its current form, it is not overtly clear what the paradox is in the first part of this manuscript. Instead, the crux of that message is only spelt out in the summary (copied below).
‘Japan invests heavily in breeding macaques for experimentation under
controlled conditions, while simultaneously capturing and killing large numbers of wild
macaques in the name of wildlife management. Japan’s situation thus fundamentally
differs from those in Europe and North America, where wild NHPs are absent, and those
in parts of Southeast Asia, where many NHP species are endangered.’

I suggest this paradox be captured and explained clearly at the start of the manuscript possibly at the end of Section 1 before going into the current situation in Japan.

Perhaps the translation of the manuscript didn’t work so well for a couple of paragraphs in the current Japan context Section 2. More details and clarity is required in relation to the statements and the explanations about the wild-caught monkeys captured in FY2020 and used for undisclosed activities in the footnote and later animals in FY2024 and how they were used.
For example, please check in relation to FY2020, ‘Most of these animals were likely euthanized in practice (Ministry of the Environment, Japan, n.d.).’

Following this pargragh, then there are details about FY2024, where wild-caught monkeys are not allowed to be used in 15 of the 29 prefectures. Please can you list all 29 prefectures and indicated which permit and which do not permit their use. Are the academic institutions following the guidelines within the prefectures that don’t permit the use of wild caught monkeys. This details needs further expansion and clarification and double checking please. If necessary, please double check these details via informing and getting feedback from relevant members of the Japan Neuroscience Society.
Are there some public records that indicate numbers of animals used in Japan and references to the legal papers.

Further, some of the historical context in Section 3 provides too much narrative based detail. Of course, some of this content is critically important and very helpful to have written down but please cut the length and the extent of the details and cite some papers for others to refer to.

Also rather than leaving it to the summary, please provide more insights and details into understanding the final point (from the summary) of the below text…
‘This contrast provides at least prima facie grounds for carefully examining whether—and to what
extent—the approach Japan ought to adopt for the sake of ethical practices in this domain
should simply mirror the strategies that may be appropriate elsewhere in the world.’
For example, please go into details earlier on about the F2 requirement for biomedical primate use across the world so readers understand this need in relation to developing the current situation. These ethical use regulations are needed for these special animals so that animal populations across the world are protected and to ensure high standards of welfare, and health and wellbeing of these special animals and the researchers.
Other countries are also grappling with this paradox of sorted but as you indicate each country’s situation is unique. In St Kitts and Nevis, they also have a problem with monkeys, but for them these are introduced from Africa vervet monkeys so there might be a comparison of sorts to make. There is no natural predators on the islands so the monkeys decimate the sugar cane and other crops of this Caribbean nation. There has also been an extensive genetics research program although it has been making use of wild caught monkeys, so they are grappling with consent issues, and the research is not supported by the F2 international regulations. There is some literature about this (e.g., overviews via these weblink to explore further if there is a comparison to be made).
https://uchri.org/awards/whose-consent-matters-scientific-experimentation-and-the-monkey-problem-of-st-kitts/
https://www.unep.org/news-and-stories/story/caribbean-wrestles-mischievous-invaders-monkeys

Minor point about the table – the years need to be written in full (e.g., 52, 53, 70 etc should be written as 1952, 1953, 1970).
What does Diet mean? Please put this

Is there a way forward to be presented at the end of the manuscript?

1st Author Response Letter

**Response to comments from Editors and Reviewers:**

**Reviewer 2’s comments**

Major Concern 1: The central premise "more animals are killed" claim is not demonstrated

The manuscript and especially the media statement imply that Japan kills more macaques because wildlife-management animals cannot be used in research. That conclusion requires evidence that wild-caught macaques would substitute for purpose-bred animals and reduce breeding or research sacrifice approximately one-for-one. The available numbers do not establish that counterfactual. Wildlife-management captures may occur regardless of research demand, and many captured animals may be unsuitable for research for a variety of reasons including unknown age and health history, infectious-disease risks, behavioral stress and difficulty acclimating, uncertain genetic or experimental characteristics, scientific-validity requirements, logistical and regulatory costs.

I suggest that the authors recast this as a question or conditional argument. For example, if appropriately screened wildlife-management animals could substitute for some purpose-bred research animals without increasing capture incentives, the current system might increase the total number of macaques killed.

Thank you for this important comment. We agree that the original manuscript stated this possibility too strongly.

We have therefore substantially revised the argument and no longer present an increase in the total number of macaques killed as an established consequence of the current regulatory system. Instead, we now frame it as a question and identify two conditions on which the answer depends: (1) whether captured wild macaques could substitute for purpose-bred research animals, and (2) whether relaxing the restriction on the experimental use of captured macaques would increase the number of animals captured. We have added the following paragraphs in the Concluding Remarks.

Does the maintenance of this restriction, which continues to limit the experimental use of wild macaques today, in fact lead to more animals being killed overall? If appropriately screened wildlife-management animals could substitute for some purpose-bred research animals without increasing capture incentives, the current system—which authorizes the euthanasia of culled wild macaques while simultaneously breeding new ones for research—might paradoxically increase the total number of macaques killed by humans, thereby conflicting with the ideal of animal welfare.

Addressing this question requires examining two largely separate issues. The first is whether captured wild macaques could substitute for purpose-bred research animals. As some medical researchers have repeatedly noted, wild macaques present several difficulties as laboratory animals: their age and health history are typically unknown, they are unaccustomed to artificial environments, and they frequently carry pathogens transmissible to humans. By contrast, Japanese macaques supplied by the NBRP—with well-documented genetic backgrounds, acclimation to laboratory conditions, and reduced biosecurity risks—are in high demand for research in fields such as neuroscience and immunology. The benefit and necessity of highly controlled breeding of NHPs in these scientific fields does not hinge on whether the experimental use of wild-caught Japanese macaques is permitted. Nevertheless, in other areas such as molecular and cellular biology, basic physiology, and certain forms of preclinical research, strict acclimation or genetic uniformity is not always the primary concern. In some lines of research, genetic diversity is considered advantageous, or greater weight may be placed on securing a sufficiently large number of analyzable individuals. If safety concerns—especially zoonotic risks—could be adequately managed, wild-caught Japanese macaques could be considered as candidates for research use in such domains.

The second issue is whether relaxing the restriction on the experimental use of captured macaques would increase the number of animals captured. As some primatologists argued, integrating culled animals into research risks creating a problematic incentive structure where scientific demand could inadvertently encourage additional captures under the guise of damage control. In recent years, however, the annual number of wild Japanese macaques captured has more than doubled relative to around 2000, reaching over 20,000, whereas the NBRP supplies only a few dozens per year for research. So long as demand for Japanese macaques as laboratory animals does not greatly exceed this scale, relaxing the restriction seems unlikely to affect the number captured. That situation could change, however, if research demand were to grow well beyond the current scale, or if a substantial decline in wild populations were to sharply reduce the number of animals captured for wildlife management.

Major Concern 2: "Ban" and "wild-caught" require more precise definitions

The paper acknowledges that Japanese law does not uniformly prohibit capturing macaques for academic research and reports 170 research-permitted captures in FY2020. The operative restriction instead appears to concern diverting macaques captured under wildlife-management authority into experimental use. The manuscript should consistently distinguish:

1. Macaques captured specifically under academic-research permits.

2. Macaques captured for damage prevention.

3. Macaques captured for population adjustment.

4. Purpose-bred research macaques.

5. Animals used in invasive experiments versus observational, ecological, veterinary, or other research.

Calling the current policy a "de facto ban on wild-caught macaques" is potentially misleading when academic-research capture remains legally possible.

Thank you for this important clarification. We agree that the original manuscript used the terms “ban” and “wild-caught” too broadly and could therefore give the misleading impression that all research use of wild-caught Japanese macaques is legally prohibited.

We have therefore revised the beginning of the Current Regulations section to explain that wild Japanese macaques may be captured through three principal and legally distinct pathways: (1) capture for damage prevention, (2) capture for population adjustment, and (3) capture under an academic-research permit. The first two categories together constitute the wildlife-management captures. The article now focuses primarily on macaques captured for wildlife management rather than on wild-caught macaques in general. More specifically, we revised the text as follows.

From:

In contemporary Japan, large-scale capture of Japanese macaques is carried out primarily by municipalities and prefectural governments for the purpose of “wildlife management,” including damage prevention (to residential environments, agriculture, forestry, fisheries, and ecosystems) and population adjustment. These activities are legally grounded in Japan’s principal wildlife statute, the Wildlife Protection and Management Act, and are implemented through two prefecture-level instruments: each prefecture’s Wildlife Protection and Management Plan and its Category II Specified Wildlife Management Plan (Ministry of the Environment, Japan, 2017). In FY2020, 21,985 Japanese macaques were captured for damage prevention and 6,062 were captured for population adjustment. Most of these animals were likely euthanized in practice (Ministry of the Environment, Japan, n.d.).

To:

In contemporary Japan, a wild Japanese macaque may be captured under three principal pathways, each legally distinct, provided by the Wildlife Protection and Management Act, the country’s principal wildlife statute: capture for damage prevention (to residential environments, agriculture, forestry, fisheries, and ecosystems), capture for population adjustment, and capture under an academic-research permit. The first two together constitute “wildlife management” and are carried out primarily by municipalities and prefectural governments through two prefecture-level instruments: each prefecture’s Wildlife Protection and Management Plan and its Category II Specified Wildlife Management Plan (Ministry of the Environment, Japan, 2017).

The third pathway, academic-research capture, is legally separate and requires case-by-case authorization. In FY2020, 166 Japanese macaques were captured for academic research purposes under prefecture-issued permits and 4 under MOE-issued permits, for a total of 170 (Ministry of the Environment, Japan, n.d.). Available public statistics do not indicate whether these animals were used in invasive laboratory experiments, in observational or ecological studies, in veterinary research, or in other forms of academic research, nor do they report their subsequent disposition. These figures should therefore not be read as evidence that all the 170 wild macaques entered biological or medical experimentation.

The present article focuses primarily on macaques captured for wildlife management, which are far more numerous than those captured under academic-research permits. In FY2020, 21,985 Japanese macaques were captured for damage prevention and 6,062 were captured for population adjustment (Ministry of the Environment, Japan, n.d.).

Major Concern 3: The historical-review methodology is insufficiently described

The manuscript does not explain systematically how documents and events were identified and selected. It should include a short methods section stating; databases, archives, government websites, and date ranges searched; search terms and inclusion criteria; how the 29 prefectural plans were identified; which 15 plans expressly prohibited experimentation or required euthanasia; whether Japanese passages were translated by the authors; how potentially conflicting historical accounts were adjudicated; whether newspaper reports were used as primary evidence or contextual sources.

A supplementary table listing all 29 prefectures, plan dates, relevant provisions, and source links would materially strengthen the paper.

Thank you for this important comment. We agree that the original manuscript did not sufficiently explain how the regulatory and historical sources were identified, selected, and evaluated. We have therefore added a new Methods section (Section 2) describing our documentary approach in detail:

2. Methods

This study combines a review of currently operative regulations with a documentary reconstruction of their historical formation. It is not a systematic review, and no single bibliographic database defines its corpus. For the current regulatory framework (Section 3), we examined the relevant Japanese statutes, materials published by the Ministry of the Environment (MOE), the prefectural Category II Specified Wildlife Management Plans for Japanese macaques (obtained from the official websites of the respective prefectural governments), guidelines issued by the relevant academic societies, and the regulations and guidelines adopted by major primate-research institutions.

For the historical reconstruction (Section 4), we drew primarily on contemporaneous sources, including statements and reports issued by academic societies and articles published in journals such as Primate Research and Experimental Animals. These materials were supplemented by later retrospective accounts and memoirs. Retrospective sources were read critically and, where accounts differed, greater evidentiary weight was given to contemporaneous documentation. We also examined newspaper coverage, particularly articles published in the Asahi Shimbun, which reported extensively on issues concerning Japanese macaques during the period examined. Articles were identified through the Asahi Shimbun Cross-Search database using combinations of Japanese-language search terms such as “Japanese macaque,” “experiment,” and “Wildlife Protection Act.” All translations from Japanese are our own.

We have also added Supplementary Table 1 listing all 29 prefectures, the current plan period, whether the plan contains an explicit statement against experimental use, whether it explicitly requires killing captured macaques, and the official prefectural webpage from which the plan was obtained.

Major Concern 4: The ethical analysis needs a more balanced framework

The discussion emphasizes numerical reduction in deaths but does not adequately compare all relevant dimensions, including; total number of animals killed, duration and intensity of suffering, stress associated with capture, transport, quarantine, and laboratory acclimation, biosecurity and zoonotic risk, scientific validity and data quality, conservation of local populations and genetic structure, the possibility that research demand could create incentives for additional capture, the 3Rs, especially replacement and reduction, public legitimacy and governance safeguards.

Using a macaque already scheduled for euthanasia might reduce breeding demand, but it could also prolong or intensify that individual animal's suffering. A defensible conclusion requires considering both numbers and welfare severity.

Thank you for this important comment. We agree that the original manuscript placed too much emphasis on the numerical question of how many macaques are ultimately killed and did not adequately consider the qualitative severity of animal suffering or the broader scientific, ecological, and governance issues involved.

In response, we have substantially expanded and restructured the ethical discussion in the Concluding Remarks. We have added the following passage:

In addition, a defensible ethical evaluation of the paradox cannot rely solely on a utilitarian calculus of numerical reduction in animal deaths; it must carefully weigh the qualitative severity of welfare burdens alongside other complex scientific and social dimensions. Utilizing a wild-caught macaque already scheduled for euthanasia under wildlife management programs might seemingly reduce breeding demand. Also, it is not always clear whether the “euthanasia” of wild-caught macaques sufficiently considers their welfare, and in this regard, well-equipped research institutions may be better placed to ensure the ultimate euthanasia is carried out appropriately. However, keeping wild-caught macaques alive for research could simultaneously prolong or intensify that individual animal’s suffering. Specifically, wild macaques subjected to capture, transport, quarantine, and laboratory acclimation may experience intense, prolonged psychological and physiological distress compared to purpose-bred animals who are habituated to controlled laboratory environments from birth. Furthermore, wild-caught primates carry substantial biosecurity and zoonotic risks (such as B virus), which can compromise scientific validity and data quality. This, in turn, risks violating the 3Rs principles—particularly reduction and refinement—by requiring larger sample sizes to overcome uncontrolled genetic and environmental variables.

Major Concern 5: The quantitative displays cannot support strong trend or causal conclusions

Figure 1 combines:

* Japanese-macaque captures reported by fiscal year.

* Imports of all nonhuman primates reported by calendar year.

* Data assembled from different sources and classification systems.

These are not directly comparable. The caption and text should emphasize their descriptive nature and explain discontinuities, missing values, category changes, and whether imported animals include species or purposes unrelated to the argument.

Table 2 also needs qualification. It reports deaths among survey respondents, not national use; "cumulative through 1977" is presented alongside annual counts, and deaths do not necessarily equal animals entering experiments. It should not be used to infer a national trend without denominators and survey-coverage information.

Thank you for this important comment. We agree that, in the original manuscript, Figure 1 and Table 2 could give a stronger impression of direct comparability, national representativeness, and causal inference than the underlying data justify. We have therefore revised both the captions and the main text to make their descriptive and limited nature explicit.

First, regarding Figure 1, we now state directly in the figure caption that the two series are not directly comparable and are not intended to demonstrate a causal relationship:

From:

Fig. 1. Temporal trends in (i) the number of wild Japanese macaques captured in Japan (reported by fiscal year) and (ii) the number of NHPs imported into Japan (reported by calendar year). Data for (i) were obtained from the Japan Hunters Association (n.d.). Data for (ii) were taken from the Committee for the Study of Experimental Primate Resources and Development (1987) for years up to 1986; from the December issues of Nihon Bōeki Geppō [Monthly Return of the Trade of Japan] for 1987–2001; and from the Ministry of Finance, Japan (n.d.) for 2002 onward (searched using HS code 0106.11).

To:

Fig. 1. Historical statistics on reported wild Japanese macaque captures and NHPs imports into Japan. Capture figures are reported by fiscal year and import figures by calendar year. The two series are not directly comparable and the figure is not intended to demonstrate a causal relationship. Import figures cover all NHPs and are not limited to macaques or animals imported for experimental purposes. Capture data were obtained from the Japan Hunters Association (n.d.). Import data were taken from the Committee for the Study of Experimental Primate Resources and Development (1987) for years up to 1986; from the December issues of Nihon Bōeki Geppō [Monthly Return of the Trade of Japan] for 1987–2001; and from the Ministry of Finance, Japan (n.d.) for 2002 onward (searched using HS code 0106.11). Differences among these sources may affect the continuity of the series.

To reinforce these limitations in the main text, we have also added the following statement:

Available Japanese statistics on NHP imports and wild Japanese macaque captures are shown in Fig. 1 as contextual information. Because the two datasets differ in temporal units, sources, coverage, and classification, no direct quantitative comparison between them is attempted here.

Second, we have substantially revised the caption of the former Table 2, now Table 1, to clarify the nature and limitations of the underlying survey data.

From:

Table 2. Annual deaths of NHPs by species at Japanese research institutions (survey respondents only).

To:

Table 1. Reported deaths of selected NHP species at Japanese research institutions, based on a 1985 questionnaire survey. Adapted from Table 11 of Minezawa et al. (1986). The survey received responses from 160 of 252 institutions selected as likely users of primates. Respondents reported deaths among primates used in their laboratories, including deaths from disease and accidents. These figures therefore represent responding institutions only and are neither national totals nor direct measures of experimental use. “1977 and earlier” is an aggregate category and is not directly comparable with the annual counts for 1978–1984.

We revised the surrounding historical interpretation so that the mortality data are no longer used by themselves to infer a national trend. The original manuscript stated that the shift toward greater use of Japanese macaques was “reflected” in the mortality data. In the revised manuscript, we instead attribute the interpretation directly to the contemporary survey authors:

Minezawa et al. (1986) reported the results of a questionnaire survey of Japanese research institutions, including the mortality data reproduced in Table 1, and concluded from the survey as a whole that the use of Japanese macaques had increased as access to rhesus macaques became more restricted.

Major Concern 6: The policy recommendation needs concrete safeguards

If the authors wish to argue that limited research use of wildlife-management animals should be considered, they should specify the conditions necessary to prevent harmful incentives. Possible safeguards include:

* Capture decisions made independently of research demand.

* No payment structure that rewards additional capture.

* Prospective veterinary and infectious-disease screening.

* Independent ethical review.

* Scientific justification that purpose-bred animals are unnecessary.

* Transparent reporting of capture reason, disposition, and research use.

* Monitoring of whether use actually reduces purpose-bred demand.

* Limits by research type and welfare burden.

Without such conditions, "context-sensitive regulation" remains too general.

Thank you for this important comment. We agree that any consideration of the research use of wildlife-management macaques would require strict safeguards. We have therefore added the following clarification to the Concluding Remarks:

A robust and defensible governance framework must therefore address all of these dimensions: balancing the overall reduction in animal deaths against the heightened severity of individual suffering, public legitimacy, and the strict requirements of biosecurity. A detailed specification of such safeguards, however, lies beyond the scope of this article.

Our intention is not to propose an immediate relaxation of the current restriction, but to emphasize that any future reconsideration would need to address these competing ethical and practical concerns. Because the article is primarily a historical analysis, we regard the detailed design of a regulatory framework as beyond its present scope.

Minor comments

Replace "overabundance" with more neutral wording unless a population-management assessment supports it.

Thank you. We have replaced the broad characterization of Japanese macaques as “overabundant” with more neutral wording. For example, the Introduction now refers to “population growth,” and the historical description of the 1999 reform now refers to populations “that had markedly increased or decreased.”

"Legally mandated wildlife-management programs" may overstate the legal position; the programs appear legally authorized and administratively implemented, not necessarily mandated in every case.

We agree and have revised the Abstract to read “legally authorized wildlife management programs.”

Correct "annal supply target" to "annual supply target."

Thank you. Corrected to “annual supply target.”

Correct "an one-size-fits-all approach" to "a one-size-fits-all approach."

Thank you. Corrected to “a one-size-fits-all approach.”

Use consistent capitalization for "Japanese macaques."

Thank you. We have standardized the term as “Japanese macaques” throughout the manuscript.

Clarify whether the 1,190 NBRP figure represents animals distributed, experiments, or transfers.

We have clarified and updated this point. The revised manuscript now states:

Since the pilot phase began in FY2006, the NBRP has distributed a total of 1,248 individual Japanese Macaques to research institutions through FY2025, corresponding to an annual mean of about 62 over 20 years (Kyoto University NBRP “Japanese Macaques,” 2026).

The manuscript is long and detailed for a short communication. Table 1 could be moved to supplementary material and the historical narrative shortened.

We agree. We have shortened the historical narrative and moved the former Table 1 to the Supplementary Material as “Supplementary Table 2.”

The graphical abstract and highlights reproduce the causal claim too strongly and should be revised with the manuscript.

The phrase "ecologists' priority" risks portraying one heterogeneous community as having a single position. More qualified language would be preferable.

We agree and have revised them. Specifically, Highlights have been revised as follows.

From:

In Japan, more than 20,000 wild macaques are culled each year due to overabundance.

However, their use in research has been de facto banned since 2001.

Its regulatory logic reflects ecologists’ priority on local population conservation.

To:

Each year Japan captures over 20,000 wild macaques, most of which are killed.

Experimental use of wildlife-management macaques is tightly restricted in Japan.

This policy reflects a conservation priority on local populations, not species.

The discussion of inadequate stakeholder participation should be supported with evidence about the actual consultation process, not inferred only from later objections.

Thank you. We have added evidence on the composition of the 1999 review committee, as shown below.

From:

When the Environment Agency solicited public comments in September 1999 on the Standards for the Eighth Wildlife Protection Plan, three board members of the Primate Society of Japan submitted an opinion statement warning against overly easy adoption of nuisance control and requested language such as: “As a general rule, live-captured individuals should not be used as experimental animals” (Nigi et al., 2000). Reflecting these views, the Agency’s draft version of the Technical Manual for Specified Wildlife Protection and Management Plans (Japanese Macaque Edition), released in 2000, stated that “captured individuals should, in principle, be euthanized” and that “capture for the purpose of experimental use is not permitted.”

To:

In July 1999, following the revision of the Wildlife Protection Act, the Environment Agency established the Committee on Wildlife Protection and Management to deliberate related guidelines and standards. Its 14 members consisted primarily of specialists in fields such as wildlife management, ecology, and nature conservation. They included a primatologist specializing in the conservation ecology of Japanese macaques who also served on the board of the Primate Society of Japan. By contrast, the committee included no physiologists, neuroscientists, or other members who could represent the interests of researchers using Japanese macaques as experimental animals (Environment Agency, Japan, 1999a). When the Environment Agency solicited public comments in September 1999 on the Standards for the Eighth Wildlife Protection Plan, three board members of the Primate Society of Japan, having obtained information from the aforementioned primatologist about the committee’s internal deliberations, submitted an opinion statement warning against overly easy adoption of nuisance control and requested language such as: “As a general rule, live-captured individuals should not be used as experimental animals” (Nigi et al., 2000).

Reflecting these views, the committee discussed limiting the forms of scientific research recommended as beneficial uses of captured animals to research directly related to wildlife protection and management, thereby excluding experimental research in fields such as medicine. The standards were subsequently revised along these lines (Environment Agency, Japan, 1999b; Yoshida, 2001). The draft version of the Technical Manual for Specified Wildlife Protection and Management Plans (Japanese Macaque Edition) subsequently released by the Environment Agency, stated that “captured individuals should, in principle, be euthanized” and that “capture for the purpose of experimental use is not permitted.”

We have also revised the relevant passage in the Concluding Remarks as follows:

From:

In particular, researchers who had relied on captured primates for experimentation were not adequately afforded opportunities to participate substantively in discussions that would shape the policy decision.

To:

The review committee convened by the Environment Agency included a board member of the Primate Society of Japan, but it included no one positioned to represent the interests of researchers who used captured macaques in experiments. Those who opposed the emerging regulation voiced their objections through written statements, yet there is no evidence that they were able to attend the Environment Agency’s meetings on the matter.

**Reviewer 2’s comments**

## Major Concern 1

In its current form, it is not overtly clear what the paradox is in the first part of this manuscript. Instead, the crux of that message is only spelt out in the summary (copied below).

‘Japan invests heavily in breeding macaques for experimentation under controlled conditions, while simultaneously capturing and killing large numbers of wild macaques in the name of wildlife management. Japan’s situation thus fundamentally differs from those in Europe and North America, where wild NHPs are absent, and those in parts of Southeast Asia, where many NHP species are endangered.’

I suggest this paradox be captured and explained clearly at the start of the manuscript possibly at the end of Section 1 before going into the current situation in Japan.

Thank you for this helpful comment. We agree that, in the original manuscript, the central paradox was not stated clearly enough at the beginning of the article. We have therefore added the following paragraphs to the end of the Introduction.

Japan therefore presents an unusual—and seemingly paradoxical—regulatory situation. Large numbers of wild Japanese macaques are captured through wildlife-management programs and subsequently euthanized, while Japanese macaques used in biological or medical research are supplied through a separate captive-breeding system. How did these two systems come to coexist? Why did Japan come to exclude macaques captured for wildlife management from experimental use while simultaneously establishing a separate breeding-based supply system for research?

This article addresses this question by first examining the current regulatory framework governing Japanese macaques captured for wildlife management and then reconstructing the historical process through which that framework emerged, with particular attention to the policy controversy around 2000.

Major Concern 2

Perhaps the translation of the manuscript didn’t work so well for a couple of paragraphs in the current Japan context Section 2. More details and clarity is required in relation to the statements and the explanations about the wild-caught monkeys captured in FY2020 and used for undisclosed activities in the footnote and later animals in FY2024 and how they were used.

For example, please check in relation to FY2020, ‘Most of these animals were likely euthanized in practice (Ministry of the Environment, Japan, n.d.).’

Following this pargragh, then there are details about FY2024, where wild-caught monkeys are not allowed to be used in 15 of the 29 prefectures. Please can you list all 29 prefectures and indicated which permit and which do not permit their use. Are the academic institutions following the guidelines within the prefectures that don’t permit the use of wild caught monkeys. This details needs further expansion and clarification and double checking please. If necessary, please double check these details via informing and getting feedback from relevant members of the Japan Neuroscience Society.

Are there some public records that indicate numbers of animals used in Japan and references to the legal papers.

Thank you for this important comment. This concern partly overlaps with Reviewer 1’s Major Concern 2 & 3, and we have substantially revised the Current Regulations section accordingly. We now distinguish clearly among capture for damage prevention, capture for population adjustment, and capture under academic-research permits. We also moved the information on academic-research capture from the former Footnote into the main text so that these distinctions are clear from the outset.

In addition, we rechecked all 29 prefectural Category II Specified Wildlife Management Plans, updated the information to August 2026, and added Supplementary Table 1 listing the relevant provisions and official prefectural sources. We have also strengthened the discussion of what happens to macaques captured for wildlife management by adding evidence from an MOE survey and recent prefectural data, while explicitly acknowledging the absence of comprehensive nationwide disposition data.

For example, we revised the following passage:

From:

Following these Guidelines, each prefecture formulates a Category II Specified Wildlife Management Plan for Japanese macaques. As of June 2025, among the 29 prefectures that have established such plans, 15 explicitly state that captured macaques must not be used for experiments and/or must be euthanized. Where capture is conducted pursuant to these plans, diversion to research is effectively precluded.

To:

Following these Guidelines, prefectures formulate Category II Specified Wildlife Management Plans for Japanese macaques. As of August 2026, 29 prefectures had established such plans. Of these, 15 explicitly state that captured macaques should or must not be used for experiments, require that they be killed, in most cases specifying euthanasia (i.e., methods that minimize suffering), or contain both types of provision (Supplementary Table 1). Where capture is conducted pursuant to these plans, diversion to research is effectively precluded.

The great majority of Japanese macaques captured for wildlife management are, we infer, killed in accordance with these Guidelines. A 2004 MOE report based on a survey of the relevant prefectures stated that captured macaques were “mostly” disposed of by killing, with only limited cases of captive holding (Ministry of the Environment, Japan, 2004). More recent prefectural data suggest that this pattern has continued: in Kanagawa Prefecture in FY2025, all 42 macaques recorded as captured for wildlife management were killed (Kanagawa Prefecture, 2026). Comprehensive nationwide data on the post-capture disposition of macaques are not currently available, but the sources that do exist strongly indicate that killing remains the predominant outcome.

Major Concern 3

Further, some of the historical context in Section 3 provides too much narrative based detail. Of course, some of this content is critically important and very helpful to have written down but please cut the length and the extent of the details and cite some papers for others to refer to.

Thank you for this helpful comment. We have revised and condensed the historical discussion so that it focuses more directly on the developments necessary to explain the emergence of the present regulatory framework.

For example, we deleted or condensed several contextual details, including the eradication of Taiwanese macaques in Wakayama, the discussion of the Nature Neuroscience editorial and the Japanese neuroscientist’s rebuttal, media coverage around 2000, and the activities of individual researchers.

## Major Concern 4

Also rather than leaving it to the summary, please provide more insights and details into understanding the final point (from the summary) of the below text…

‘This contrast provides at least prima facie grounds for carefully examining whether—and to what extent—the approach Japan ought to adopt for the sake of ethical practices in this domain should simply mirror the strategies that may be appropriate elsewhere in the world.’

For example, please go into details earlier on about the F2 requirement for biomedical primate use across the world so readers understand this need in relation to developing the current situation. These ethical use regulations are needed for these special animals so that animal populations across the world are protected and to ensure high standards of welfare, and health and wellbeing of these special animals and the researchers.

Thank you for this important suggestion. We have revised both the Introduction and the Concluding Remarks to make the international regulatory background and our comparative argument clearer.

First, we added a concrete example of the international regulatory framework in the Introduction:

For example, the EU prohibits the use of wild-caught animals in scientific procedures in principle and requires NHPs used in research to be captive-bred or sourced from self-sustaining colonies, subject to scientifically justified exceptions (European Parliament and Council of the European Union, 2010, Arts. 9–10).

We also clarified in the Concluding Remarks that our argument is not intended to question the importance of such strict regulations in other regions, but rather to consider whether the same formal approach is necessarily the most appropriate under Japan’s different ecological and historical conditions:

Then, to respect the welfare of NHPs more comprehensively, it might be preferable for Japan to adopt regulations that differ from the current ones, which are, on the surface, similar to international standards. This suggestion by no means negates the importance of the strict regulations governing the use of wild macaques for research in other regions, such as the EU. On the contrary, it intends to apply their underlying principle of respect for animal welfare within a different natural and historical context.

Major Concern 5

Other countries are also grappling with this paradox of sorted but as you indicate each country’s situation is unique. In St Kitts and Nevis, they also have a problem with monkeys, but for them these are introduced from Africa vervet monkeys so there might be a comparison of sorts to make. There is no natural predators on the islands so the monkeys decimate the sugar cane and other crops of this Caribbean nation. There has also been an extensive genetics research program although it has been making use of wild caught monkeys, so they are grappling with consent issues, and the research is not supported by the F2 international regulations.

Thank you for this valuable suggestion. Following the reviewer’s suggestion, we added a brief comparison with St. Kitts to the Concluding Remarks. We use this case not as a direct analogue to Japan, but as an example showing that similar tensions between wildlife management, agriculture, animal welfare, and biomedical research can arise under very different local conditions.

Similarly, in countries other than Japan, respect for animal welfare might call for regulatory approaches alternative to the existing ones. For example, on St. Kitts, African green monkeys (Chlorocebus sabaeus)—transported from West Africa during the colonial slave trade, free of natural predators, and now numbering on the order of the island’s human population—are widely regarded as an invasive agricultural pest, yet are simultaneously valued as a tourist attraction. And, in sharp contrast to Japan, they are captured in the wild and used in biomedical experiments—both on the island and, through export, in laboratories abroad (Gallagher et al., 2022). Given this highly specific and complex situation, it would be an urgent issue to consider what can and should be done to ensure the welfare of NHPs.

Minor comments

Minor point about the table – the years need to be written in full (e.g., 52, 53, 70 etc should be written as 1952, 1953, 1970).

Thank you. We have corrected the year format throughout the chronological table.

What does Diet mean? Please put this

Thank you. We have clarified this term at its first relevant occurrence in the main text by writing “the National Diet (Japan’s parliament).”

Is there a way forward to be presented at the end of the manuscript?

Thank you for this important suggestion. The primary aim of this article is to reconstruct the historical and regulatory background of the present Japanese system, rather than to design a detailed alternative regulatory framework. A fully developed policy proposal would therefore go beyond the scope of the present article.

Nevertheless, we have revised the Concluding Remarks to indicate a possible direction forward. We now emphasize that any reconsideration of the current system would need to weigh not only the total number of animals killed, but also individual suffering, biosecurity, scientific validity, conservation, public legitimacy, and the risk that research demand could encourage additional capture. On this basis, we suggest that future NHP regulation should be context-sensitive rather than assuming that identical formal rules are necessarily appropriate under different ecological and historical conditions.

We hope that these revisions provide a clearer indication of a possible way forward while remaining consistent with the historical and analytical purpose of the article.

Accept Letter

Dear Prof Sawai,   
 
Thank you for submitting your manuscript to Current Research in Neurobiology.

I am pleased to inform you that your manuscript has been accepted for publication. Congratulations.

My comments, and any reviewer comments, are below.
    
Your accepted manuscript will now be transferred to our production department. We will create a proof which you will be asked to check, and you will also be asked to complete a number of online forms required for publication. If we need additional information from you during the production process, we will contact you directly.

We appreciate and value your contribution to Current Research in Neurobiology. We regularly invite authors of recently published manuscript to participate in the peer review process. If you were not already part of the journal’s reviewer pool, you have now been added to it. We look forward to your continued participation in our journal, and we hope you will consider us again for future submissions.

We would also like to invite you to take part in our CRNEUR Author [Question & Answer (Q&A)](https://www.elsevier.com/__data/promis_misc/QA_Authors.docx), which could get published alongside your article and help to promote it. We suspect you might have an interesting story of perseverance or team work that was required for the research study to complete, or a diversity of perspectives that you might share, as a way of inspiring others about neuroscience.

Kind regards,

Anna S Mitchell, Ph.D.  
Editor in Chief  
Current Research in Neurobiology  
  
Editor and Reviewer comments:    
Both reviewers agree that the authors addressed their comments well, and that the revisions have substantially improved the paper’s clarity and impact.

Reviewer 1: Thank you for your responses to my comments. I believe the paper is much improved.

*-------- End of Review Comments --------*
