# Supplementary material for: Japan's regulations on the experimental use of wild-caught Japanese macaques and its historical context
Source: Curr Res Neurobiol. 2026 Oct 25;11:100171. doi: 10.1016/j.crneur.2026.100171 (PMC13742342; doi:10.1016/j.crneur.2026.100171)
Supplement: Supplementary file 3 [file mmc3.docx]

**Supplementary Table 1. Provisions concerning the experimental use and killing of captured Japanese macaques in prefectural Category II Specified Wildlife Management Plans, as of August 2026.** “Yes” in the second column indicates that the current prefectural management plan explicitly states that wildlife-management Japanese macaques should not be used for experimental purposes. “Yes” in the third column indicates that the plan explicitly requires captured macaques to be killed, including provisions requiring euthanasia. “No*” indicates that the plan requires captured macaques to be “disposed of” or “handled” by methods that minimize pain, but do not explicitly state that the animals are to be killed or euthanized. “No” indicates only that no such explicit provision was identified in the current plan; it does not necessarily mean that experimental use is permitted.

| Prefecture | Explicit statement against experimental use | Explicit requirement to kill/euthanize captured macaques | Current plan period | Official prefectural webpage |
| --- | --- | --- | --- | --- |
| Aomori | Yes | No* | 1 Apr 2022–31 Mar 2027 | https://www.pref.aomori.lg.jp/soshiki/kankyo/shizen/kakushuchoujuukeikaku.html |
| Miyagi | Yes | No* | 1 Apr 2022–31 Mar 2027 | https://www.pref.miyagi.jp/soshiki/sizenhogo/nihonzaru-kanrikeikaku.html |
| Akita | Yes | Yes | 1 Apr 2022–31 Mar 2027 | https://www.pref.akita.lg.jp/pages/archive/64249 |
| Yamagata | No | Yes | 1 Apr 2022–31 Mar 2027 | https://www.pref.yamagata.jp/050011/kurashi/shizen/seibutsu/choujuuhogo/hogokanri.html |
| Fukushima | No | No | 1 Apr 2022–31 Mar 2027 | https://www.pref.fukushima.lg.jp/sec/16035b/yaseityouyuuhogokannrijigyoukeikaku-fukushima.html |
| Tochigi | No | No | 1 Apr 2022–31 Mar 2027 | https://www.pref.tochigi.lg.jp/d04/eco/shizenkankyou/shizen/saruhogokannri3.html |
| Gunma | No | No | 1 Apr 2022–31 Mar 2027 | https://www.pref.gunma.jp/page/7128.html |
| Chiba | Yes | No* | 1 Apr 2022–31 Mar 2027 | https://www.pref.chiba.lg.jp/shizen/choujuu/jigyoukeikaku/keikaku.html |
| Kanagawa | Yes | Yes | 1 Apr 2023–31 Mar 2027 | https://www.pref.kanagawa.jp/docs/t4i/cnt/f986/documents/saru.html |
| Niigata | Yes | No* | 1 Apr 2022–31 Mar 2027 | https://www.pref.niigata.lg.jp/sec/kankyotaisaku/1329339654302.html |
| Toyama | No | Yes | 1 Apr 2022–31 Mar 2028 | https://www.pref.toyama.jp/1709/kurashi/kankyoushizen/shizen/kanrikeikaku/kj00017573.html |
| Ishikawa | No | No | 1 Apr 2022–31 Mar 2027 | https://www.pref.ishikawa.lg.jp/sizen/hogokanri/hogokanri.html |
| Fukui | No | Yes | 1 Apr 2022–31 Mar 2027 | https://www.pref.fukui.lg.jp/doc/021500/tokuteikeikaku/tokutei.html |
| Yamanashi | Yes | Yes | 1 Apr 2022–31 Mar 2027 | https://www.pref.yamanashi.jp/shizen/11689186440.html |
| Nagano | Yes | Yes | 1 Apr 2024–31 Mar 2029 | https://www.pref.nagano.lg.jp/yasei/sangyo/ringyo/choju/hogo/saru_5th.html |
| Gifu | No | No | 1 Apr 2023–31 Mar 2028 | https://www.pref.gifu.lg.jp/page/4783.html |
| Aichi | No | No | 1 Apr 2022–31 Mar 2027 | https://www.pref.aichi.jp/soshiki/shizen/chouju2shu.html |
| Mie | No | Yes | 1 Apr 2022–31 Mar 2027 | https://www.pref.mie.lg.jp/SHINRIN/HP/mori/000126727.htm |
| Shiga | Yes | Yes | 1 Apr 2024–31 Mar 2029 | https://www.pref.shiga.lg.jp/ippan/kankyoshizen/shizen/304225.html |
| Kyoto | No | No | 1 Apr 2022–31 Mar 2027 | https://www.pref.kyoto.jp/choujyu/tokuteichoujyu-hogokanri.html |
| Hyogo | No | No | 1 Apr 2022–31 Mar 2027 | https://web.pref.hyogo.lg.jp/nk27/12jityojyuhogokanrikeikaku.html |
| Nara | No | No | 1 Apr 2026–31 Mar 2031 | https://www.pref.nara.lg.jp/n118/67004.html |
| Wakayama | No | Yes | 1 Apr 2022–31 Mar 2027 | https://www.pref.wakayama.lg.jp/prefg/072000/23kousin/d00216365.html |
| Okayama | No | No | 1 Apr 2022–31 Mar 2027 | https://www.pref.okayama.jp/page/604310.html |
| Yamaguchi | No | No | 1 Apr 2022–31 Mar 2027 | https://www.pref.yamaguchi.lg.jp/soshiki/41/20670.html |
| Tokushima | No | No* | 1 Apr 2022–31 Mar 2027 | https://www.pref.tokushima.lg.jp/ippannokata/kurashi/shizen/7204764/ |
| Kagawa | No | No | 1 Apr 2022–31 Mar 2027 | https://www.pref.kagawa.lg.jp/midorihozen/shuryo/chojugai/index.html |
| Ehime | No | No | 1 Apr 2022–31 Mar 2027 | https://www.pref.ehime.jp/page/17935.html |
| Miyazaki | Yes | Yes | 1 Apr 2022–31 Mar 2027 | https://www.pref.miyazaki.lg.jp/shizen/kense/kekaku/20220210091423.html |

**Supplementary Table 2. Timeline of events related to the issue of experimental use of wild Japanese macaques.**

|  | National Diet / Ministries | Academic Societies / Scientists | Society |
| --- | --- | --- | --- |
| 1948 |  | ・Imanishi’s group began field research on wild Japanese macaques. |  |
| 1952 |  | ・Imanishi’s group succeeded in provisioning wild Japanese macaques at Kōjima. |  |
| 1953 |  |  | ・Opening of Takasakiyama Monkey Park; the “monkey park” boom began. |
| 1970 |  |  | ・The number of monkey parks reached a peak of 35 nationwide. |
| 1985 |  | ・Establishment of the Primate Society of Japan |  |
| 1986 |  | ・The Primate Society of Japan: *The Guiding Principles for Animal Experiments Using Nonhuman Primates* |  |
| 1997 | ・A group of Diet members submitted a proposal to revise the Wildlife Protection Act to the Environment Agency. |  |  |
| 1999 | ・Revision of the Wildlife Protection Act | ・Three board members of the Primate Society of Japan submitted an opinion statement to the Environment Agency. | ・Media reports on the false-reporting scandal at Takasakiyama Monkey Park |
| 2000 | ・The Environment Agency: the draft version of *Technical Manual for Specified Wildlife Protection and Management Plans (Japanese Macaque Edition)*  ・The Environment Agency: the final version of *Technical Manual for Specified Wildlife Protection and Management Plans (Japanese Macaque Edition)* | ・The Physiological Society of Japan submitted three opinion statements to the Environment Agency.  ・The Primate Society of Japan issued a position statement. | ・Media reports on illegal trafficking of Japanese macaques by unlicensed dealers |
| 2001 |  | ・The Primate Society of Japan requested Wakayama Prefecture to euthanize Taiwanese macaques.  ・The Physiological Society of Japan and others requested the Ministry of Education, Culture, Sports, Science and Technology (MEXT) to establish a breeding-based supply system. |  |
| 2002 | ・MEXT launched the National BioResource Project (NBRP). |  |  |
| 2003 |  |  | ・Protest movement opposing the NBRP’s plan to obtain Japanese macaques from zoos and similar facilities |
| 2006 |  | ・NBRP began supplying Japanese macaques for research use. |  |
| 2013 |  | ・The Primate Society of Japan: *Guidelines for the Care and Use of Laboratory Nonhuman Primates* |  |
